# Importance of Early Detecting the Existence of Insanity

**Published:** 1853-07-01

**Authors:** 


### IMPORTANCE OF EARLY DETECTING THE EXISTENCE OF INSANITY.

Every day's experience convinces me that insanity often exists for months, and even for years, quite unsuspected by friends. I have, at this present, two cases under treatment, in each of which no cross-examination could elicit from the relatives who accompanied the patients to the asylum, an admission that they had observed any aberration of mind for more than ten days in the one, and three weeks in the other; yet in each, I was before long put in possession of abundant evidence that insanity was of fifteen months' standing in the former, and upwards of three years in the latter. Ignorant of the real duration of the disease, and of the influence of duration on the prospects of cure, the too sanguine friends of each flattered themselves that a few weeks of treatment would suffice to restore them to health. They are doomed to a bitter disappointment. In the one case, I see no prospect of cure at all, in the other there is hope, but at best, a very long period must elapse before recovery can take place. Now, had the subject of insanity been better understood by those who had the charge of these two persons, who were both young, and of uncommon intellectual powers, I feel convinced that the disease might, in each case, have been nipped in the bud, and an incalculable amount of suffering to friends and loss to society might have been spared.

The existence or occurrence of any marked peculiarity of mental or moral manifestation, or of any sudden change of habits, especially if connected with hereditary predisposition, previous attacks, suppressed or excessive discharges—whether *natural*, as menstrual, lochial, or lacteal, or merely *habitual*, as from piles, ulcers, issues or setons—repelled eruptions, prolonged nursing, want of sleep, excessive nervous excitability, or very unstable character, should at once arouse the fears and vigilance of both physicians and friends. The claims of the community are of scarcely less weight than those of the family. For, whereas, in a large proportion of such cases, it is altogether practicable, by timely and appropriate moral and physical treatment, to prevent the development of confirmed insanity; individuals are thereby spared a vast amount of suffering, and the burden thrown on the State is proportionably diminished.

Nor should we overlook the claims of posterity. Unquestionably it is as much the duty of a man to bequeath mental and physical health to his children as wealth. But and if he follow a vicious course of life, his children are apt to inherit the same tastes in an exaggerated degree; if the habitual and unrestrained indulgence of his appetites and passions leads to insanity, the sins of the father are likely to be visited on the offspring, to more than the third or fourth generation. Surely then the minister, the physician, and the friend, will not hesitate, by timely and well-timed warnings, by friendly counsels, and by all the copious resources of the art medical, to ward off the danger while yet the day is, to crush the seed ere yet the baneful plant shall germinate, and haply to make thousands yet unborn their debtors. Nor would the gratitude of contemporaries be unearned. The morality of the community is more affected by the amount of insanity in it, and of *predisposition to insanity*, than is supposed. Prevention is better than cure, and therefore I have endeavoured to draw a more especial attention to that subject.

A word on *recurrence of insanity*. People cured of insanity are of course liable to another attack, like people cured of a pneumonia or an intermittent, if again exposed to the action of the exciting causes; and yet, on returning home, the former are often permitted by their friends to resume at once the very practices which first made them insane; to attend enthusiastic, exciting, protracted religious and political meetings, keep late hours, labour too hard, expose themselves too much, indulge to excess in alcoholic drinks, strong coffee, tea and tobacco. A judicious and friendly physician might, by his timely interference, often prevent the bad consequences which must otherwise ensue, by warning, threatening, counselling, if need be, coaxing, strengthening good resolutions, and *carefully repairing physical dilapidations*, for to these latter are often to be traced a recurrence to habits of indulgence and excess.—*From the Report of the Ohio Lunatic Asylum, U. S., America.*

---

